# Effects of Health Literacy Intervention on Health Literacy Level and Glucolipid Metabolism of Diabetic Patients in Mainland China: A Systematic Review and Meta-Analysis

**DOI:** 10.1155/2021/1503446

**Published:** 2021-12-30

**Authors:** Yangli Chen, Xue Ran, Yalan Chen, Kui Jiang

**Affiliations:** Department of Medical Informatics, Medical School of Nantong University, Nantong 226001, China

## Abstract

**Objective:**

To systematically evaluate the effects of health literacy intervention on health literacy level and glycolipid metabolism of people with diabetes in mainland China.

**Methods:**

A systematic review of journal articles discussing diabetes and health literacy was performed by searching PubMed, Embase, the Science Citation Index Expanded (SCIE) database of Web of Science, the China National Knowledge Infrastructure (CNKI) database, the Chinese Scientific and Technical Journals database (CQVIP), and the Wanfang database. Cochrane Effective Practice and Organization of Care Review Group (EPOC) standards were applied for quality assessment. A meta-analysis was performed using Stata 12.0 software.

**Results:**

A total of 44 articles, including seven controlled before-and-after trials (CBAs), 27 randomized controlled trials (RCTs), and 10 nonrandomized controlled trials (non-RCTs), were included. The results showed that (1) health literacy level in the intervention group was improved compared with the preintervention and the control group; (2) fasting plasma glucose (FPG) (standardized mean difference (SMD) = −1.85, 95% CI: −2.28, −1.42), 2-hour plasma glucose (2hPG) (SMD = −2.18, 95% CI: −2.68, −1.68), and HbA1c (weighted mean difference (WMD) = −1.21, 95% CI: −1.48, −0.94) were significantly reduced in the intervention group; (3) total cholesterol (TC) (WMD = −0.43, 95% CI: −0.64, −0.23) was significantly reduced in the intervention group, although there were no statistically significant differences for triglycerides (TG) (WMD = −0.34, 95% CI: −0.73, 0.05), low-density lipoprotein cholesterol (LDL-C) (WMD = −0.20, 95% CI: −0.46, 0.07), or high-density lipoprotein cholesterol (HDL-C) (WMD = −0.06, 95% CI: −0.29, 0.17).

**Conclusion:**

Intervention based on health literacy can effectively improve health literacy levels and reduce glucose metabolism and TC level among people with diabetes mellitus, although it has no significant effect on TG, LDL-C, or HDL-C.

## 1. Introduction

According to the latest global diabetes map released by the International Diabetes Federation (IDF), approximately 463 million adults worldwide were diagnosed with diabetes in 2019. China had the largest population of people with diabetes, at approximately 116.4 million people [1]. Diabetes has become a major chronic disease impacting human health and bearing a considerable health and economic burden on society.

Diabetes health education (HE) has always been recognized as the cornerstone of effective diabetes management [2], while health literacy (HL) is an important component of diabetes HE and health promotion theories. Diabetes HL refers to an individual's ability to acquire, process, and understand diabetes-related information and medical services [3], which include the ability to read, comprehend, calculate, and utilize basic diabetes-related health information [4–6].

Interventions for people with diabetes with low HL have been implemented in Europe and America. After introducing HL intervention in 250 patients with type 2 diabetes, Kim et al. demonstrated outstanding reductions in glycated hemoglobin (HbA1c) and statistically significant improvement in patient-reported measures of diabetes burden and quality of life in the intervention group [7]. Chamany et al. found that HbA1c was significantly lower in the intervention group than the control group after implementation of diabetes self-management and telephone intervention. Both groups experienced similar improvements in self-care activities, medication adherence, and intensification [8]. This research area has attracted enduring interest in mainland China, and several studies have explored the effects of HL interventions, although the results are inconsistent. For instance, Ouyang and Liang found that levels of HbA1c and fasting plasma glucose (FPG) were significantly reduced after personalized HE intervention, while the difference in blood lipid level was not statistically significant [9]. In contrast, research from Xu et al. showed that a community–hospital–family model of intervention could effectively control the blood sugar and blood lipids of people with diabetes and improve their HL [10].

Therefore, we conducted a systematic review (SR) to analyze published studies on HL intervention for people with diabetes in mainland China in order to summarize the effects on HL level and the glucolipid metabolism of patients. This research is expected to suggest new approaches for the implementation of diabetes intervention trials. From a broader perspective, our study could be used for reference in carrying out diabetes HL interventions in other countries.

## 2. Materials and Methods

### 2.1. Research Design

This study was conducted according to Preferred Reporting Items for Systematic Reviews and Meta-Analyses (PRISMA) 2020 statement [11] (Table S1).

### 2.2. Data Sources and Searches

PubMed, Embase, the Science Citation Index Expanded (SCIE) database of Web of Science, the China National Knowledge Infrastructure (CNKI) database, the Chinese Scientific and Technical Journals database (CQVIP), and the Wanfang database were used to search for journal articles about diabetes and HL published in English or Chinese from 2010 to 2021. The following search terms in Chinese and English were used: (diabetes mellitus OR dm OR diabetes OR diabetic mellitus OR diabetic OR mellitus) AND health literacy. We updated the search on March 30, 2021. The detailed search strategy for each database is shown in Table S2.

### 2.3. Study Selection

Two investigators assessed the potential studies independently according to predefined inclusion and exclusion criteria. Conflicting decisions were addressed by negotiation or through the further judgment of a third investigator.

Inclusion criteria were as follows: (1) intervention study; (2) subjects were people with diabetes in mainland China; (3) the intervention group received HL intervention, while the control group received routine care (or the study had no control); and (4) blood glucose and lipid levels were examined and reported, as well as the HL score.

Exclusion criteria were as follows: (1) subjects had serious complications such as diabetic nephropathy, (2) not an intervention study, (3) study outcome did not include data analysis or complete data, and (4) duplicate publications or similar studies published by the same research group.

### 2.4. Data Extraction and Quality Assessment

The following data were extracted from selected studies: (1) study characteristics (e.g., author, year of publication, and intervention procedures); (2) measured blood glucose and blood lipid levels (e.g., FPG, 2-hour plasma glucose (2hPG), and total cholesterol (TC)); and (3) information on the HL scale (e.g., domains and scores).

Cochrane Effective Practice and Organization of Care Review Group (EPOC) standards [12] were applied for quality assessment including (1) random sequence generation, (2) allocation concealment, (3) baseline measurement criteria, (4) baseline characteristics before intervention, (5) data comprehensiveness, (6) blind implementation of outcome measurement, (7) protection against contamination, (8) selective reporting, and (9) other risks of bias. “Yes” (low-bias risk), “Unclear,” or “No” (high-bias risk) was assigned according to these nine considerations. The Grading of Recommendations Assessment, Development, and Evaluation (GRADE) approach was used to assess the quality of evidence for each outcome indicator [13].

### 2.5. Data Synthesis and Analysis

A meta-analysis was performed by using Stata (version 12.0). Heterogeneity among the included studies was evaluated using the *Q* test and quantified using *I*^2^. Studies were considered to be homogeneous if *I*^2^ < 50% and *P* > 0.1, and then, a fixed effect model was used to calculate the effect size. In contrast, if studies were considered to be heterogeneous, a random effect model was used to estimate the effect size. For continuous variables, the weighted mean difference (WMD) and the standardized mean difference (SMD) were applied. For all analyses, a two-tailed *P* value < 0.05 indicated statistical significance. A sensitivity analysis was performed to validate the stability of the outcomes, and funnel plots were used to identify potential publication bias.

## 3. Results

### 3.1. Search Results and Study Characteristics

**Figure 1:**
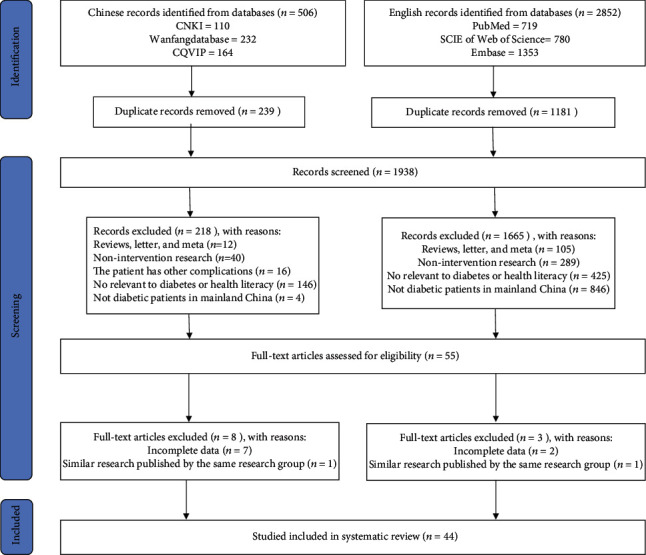
Article selection process.

A total of 3358 related studies were retrieved from the six databases, and 1938 remained after eliminating duplicates; 1883 papers were excluded following a review of the titles and abstracts of the papers. Ultimately, 44 papers [9, 10, 14–55] were eligible for inclusion, of which 41 were in Chinese [9, 10, 14–23, 25–33, 35–44, 46–55] and three were in English [24, 34, 45]. The flow of the selection process followed PRISMA guidelines and is shown in Figure 1.

Details of all included studies and the characteristics of the HL interventions, such as intervention methods and indicators, are summarized in Table 1.

### 3.2. Quality Assessment

Because it is difficult to apply a double-blind method for HL interventions and because the outcome indicators were objective endpoints, it was assumed that a single-blind evaluation was adopted in all studies. It is clearly stated in EPCO standards that allocation concealment for controlled before-and-after trial (CBA) studies should be scored “high risk” [12]. Among the included randomized controlled trial (RCT) studies, 22.2% did not specify the randomization method. None of the included studies described whether steps were taken to prevent data contamination. Overall, lack of random sequence generation, allocation concealment, and protection against contamination were the main sources of bias. A quality assessment of the included studies found low-bias risk scores over the nine areas ranging from 5 to 8, indicating a medium overall quality evaluation (Table 2).

### 3.3. Overall Effect of Interventions on HL

A total of 31 studies [9, 10, 14, 15, 17, 19–21, 25–29, 31–33, 35–38, 41–44, 46–50, 52, 55] used different HL assessment tools to analyze and report the impact of HL interventions on patient HL levels. As shown in Figure S1, self-designed questionnaires were utilized by about half of the studies [14, 17, 20, 21, 26, 28, 35, 37, 38, 41, 46, 48, 49, 52, 55] and the Diabetes Health Literacy Assessment Tool designed by Miyong Kim was employed by one-quarter of the studies [9, 10, 27, 32, 43, 44, 47]. Improving HL scores was regarded as an effective indicator by all the scales. Detailed evaluation of the contents of all assessment tools is shown in Table S3.

In terms of differences between the intervention and control groups after HL intervention, 20 studies [9, 10, 14, 15, 17, 19–21, 25–27, 29, 31–33, 35, 36, 38, 41, 52] showed that the total HL score of the intervention group was higher than that of the control group (*P* < 0.05), while two studies [28, 37] reported that the acceptability of HL of the intervention group was significantly better than that of the control group (*P* < 0.05).

In terms of pre- and postintervention changes in the intervention group, one study [43] found that there were significant differences in reading and comprehension skills (*P* < 0.05) before and after the intervention, except for numeracy skills (*P* > 0.05). Another study [50] indicated that after intervention, the proportion of patients with HL was higher than before (*P* < 0.05), while three studies [42, 48, 49] suggested that the HL level of people with diabetes was notably increased compared with preintervention. Finally, four studies [10, 21, 37, 55] showed that the excellent rate of knowledge HL was significantly boosted after the HL intervention (*P* < 0.05).

Taking these findings into consideration, we conclude that HL intervention has a significant promoting effect on general HL level in people with diabetes.

### 3.4. Meta-Analysis of Some Effect Indicators

A meta-analysis of the outcomes relating to glycemic indicators (FPG, 2hPG, and HbA1c) and lipid indicators (TC, TG (triglycerides), LDL-C (low-density lipoprotein cholesterol), and HDL-C (high-density lipoprotein cholesterol)) was performed, and the results showed a high heterogeneity in the included studies. Because our analysis includes different types of study design, a subgroup analysis was performed of each indicator according to study design type. The results revealed little variation in heterogeneity, indicating that study design type was not the main cause of high heterogeneity.

#### 3.4.1. Glucose Metabolism

**Figure 2:**
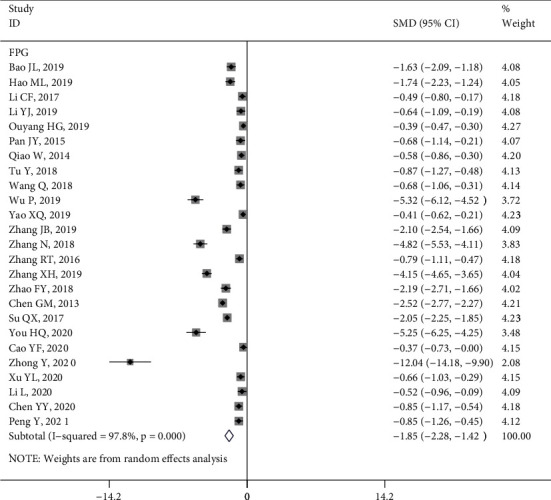
Forest plot of the effect of health literacy intervention on FPG.

*(1) FPG*. The effect of intervention on FPG level was reported in 25 studies [9, 10, 15, 16, 18, 21–23, 25–28, 30, 31, 33, 35, 37–39, 42, 43, 47, 51, 53, 54], with results showing that the intervention group is better than the control group in reducing FPG in patients (SMD = −1.85, 95% CI (−2.28, −1.42), *P* < 0.05; *Q* statistic, *I*^2^ = 97.8%, *P* < 0.1) (Figure 2). The subgroup analysis also showed that different types of study design can effectively reduce FPG levels of patients in the intervention group (Figure S2).

**Figure 3:**
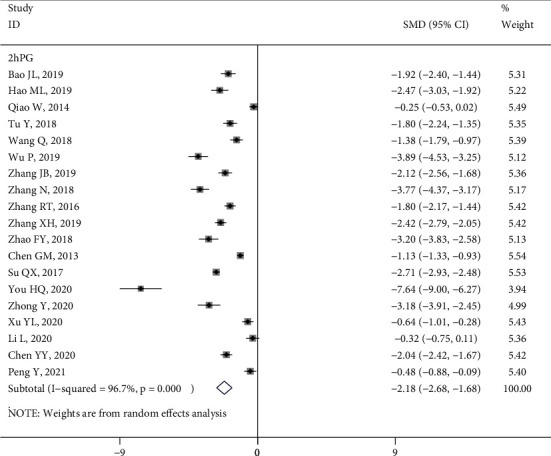
Forest plot of the effect of health literacy intervention on 2hPG.

*(2) 2hPG*. Nineteen studies [10, 15, 16, 18, 21, 22, 25, 26, 28, 31, 33, 35, 37–39, 42, 47, 53, 54] presented data regarding the effect of HL intervention on 2hPG levels, showing a significant improvement in 2hPG in the intervention group compared with the control group (SMD = −2.18, 95% CI (−2.68, −1.68), *P* < 0.05; *Q* statistic, *I*^2^ = 96.7%, *P* < 0.1) (Figure 3). However, a subgroup analysis of two non-RCT studies [16, 53] showed that the HL interventions failed to significantly improve the 2hPG levels of patients, as shown in Figure S3.

**Figure 4:**
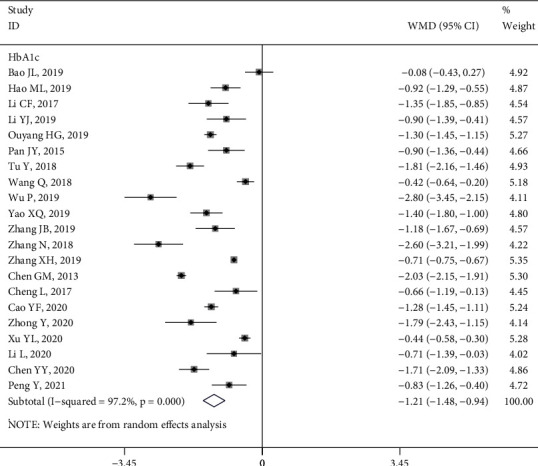
Forest plot of the effect of health literacy intervention on HbA1c.

*(3) HbA1c*. A total of 23 studies [9, 10, 15, 16, 21–28, 30, 31, 33, 34, 37–39, 43, 45, 51, 54] evaluated the impact of HL intervention on HbA1c levels. The HbA1c trend-changing graphs of two studies [24, 45] indicated that the improvement of HbA1c in the intervention group was better than that in the control group (*P* < 0.05). A meta-analysis of the relevant data in the other 21 studies showed heterogeneity *I*^2^ = 97.2%, and the results of a random effect model revealed that the intervention group achieved better HbA1c control than the control group (WMD = −1.21, 95% CI (−1.48, −0.94), *P* < 0.05) (Figure 4). The same results were also demonstrated in the subgroup analysis (Figure S4).

#### 3.4.2. Lipid Metabolism

**Figure 5:**
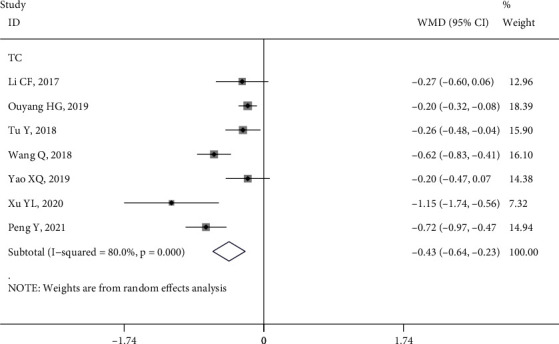
Forest plot of the effect of health literacy intervention on TC.

*(1) TC*. Seven studies [9, 10, 15, 27, 38, 39, 43] included an analysis of the effect of HL intervention on TC levels. The results of the forest map showed that TC levels were significantly improved after HL intervention (WMD = −0.43, 95% CI (−0.64, −0.23), *P* < 0.05; *Q* statistic, *I*^2^ = 80%, *P* < 0.1) (Figure 5). The results are identical in the subgroup analyses (Figure S5).

**Figure 6:**
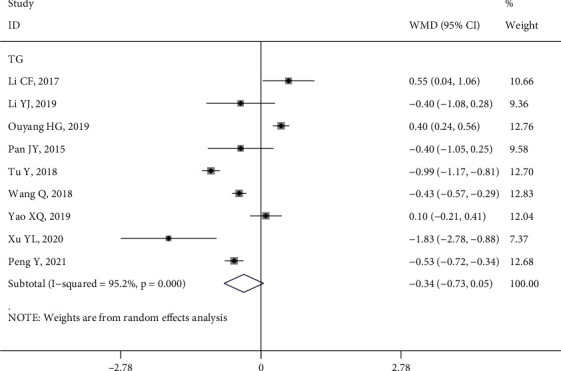
Forest plot of the effect of health literacy intervention on TG.

*(2) TG*. Nine studies [9, 10, 15, 27, 30, 39, 43, 51, 53] reported the impact of HL intervention on TG levels. The random effects model analysis showed that the improvement of TG in the HL intervention group was not statistically significant compared with the control group (WMD = −0.34, 95% CI (−0.73, 0.05), *P* > 0.05; *Q* statistic, *I*^2^ = 95.20%, *P* < 0.1) (Figure 6). However, a subgroup analysis of the RCT studies [10, 15, 27, 38, 39, 43] indicated that the HL intervention in the intervention group was effective in reducing the TG levels of patients compared with the control group (WMD = −0.44, 95% CI (−0.82, −0.07), *P* < 0.05), as shown in Figure S6.

**Figure 7:**
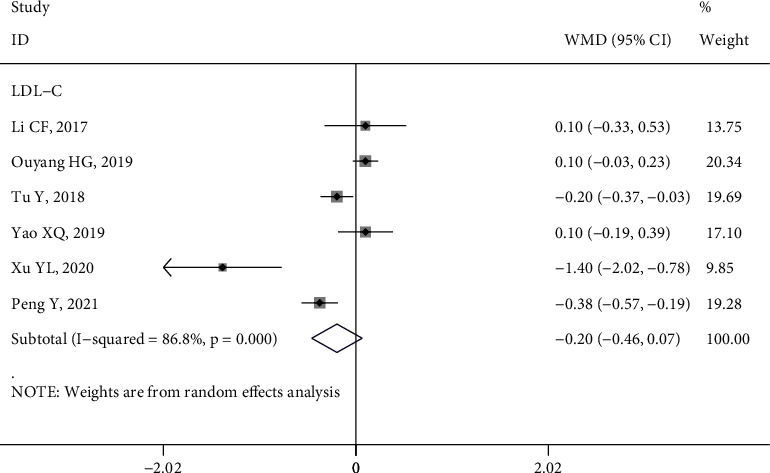
Forest plot of the effect of health literacy intervention on LDL-C.

*(3) LDL-C*. Six studies [9, 10, 15, 27, 39, 43] analyzed the impact of HL intervention on LDL-C levels (*I*^2^ = 86.8%, *P* < 0.1). The combined analysis and subgroup analysis performed by a random effect model all showed that there were no significant changes in LDL-C levels before and after HL intervention (WMD = −0.20, 95% CI (−0.46, 0.07), *P* > 0.05) (Figure 7 and Figure S7).

**Figure 8:**
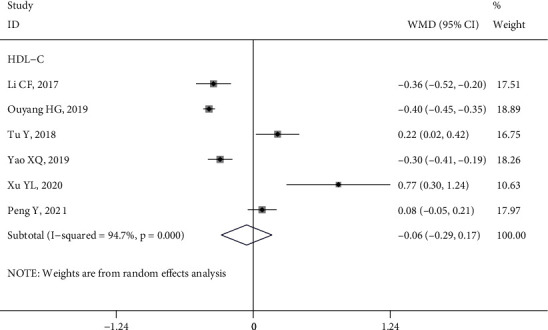
Forest plot of the effect of health literacy intervention on HDL-C.

*(4) HDL-C*. Six studies [9, 10, 15, 27, 39, 43] reported the impact of HL intervention on HDL-C (*I*^2^ = 94.7%, *P* < 0.1). The parameters extracted from analysis using a random effect model indicated that HDL-C was not significantly improved by HL intervention (WMD = −0.06, 95% CI (−0.29, 0.17), *P* > 0.05) (Figure 8). However, one non-RCT study [9] showed that the HDL-C level of the control group was better than that of the intervention group after the intervention (Figure S8).

### 3.5. Sensitivity Analysis

In order to ensure the stability of the meta-analysis conclusions, a sensitivity analysis was performed for each indicator using Stata software. After removing any one study from each indicator, the results of the meta-analyses showed that the overall effect values of each indicator did not significantly skew the conclusions (Figure S9).

### 3.6. Publication Bias

**Figure 9:**
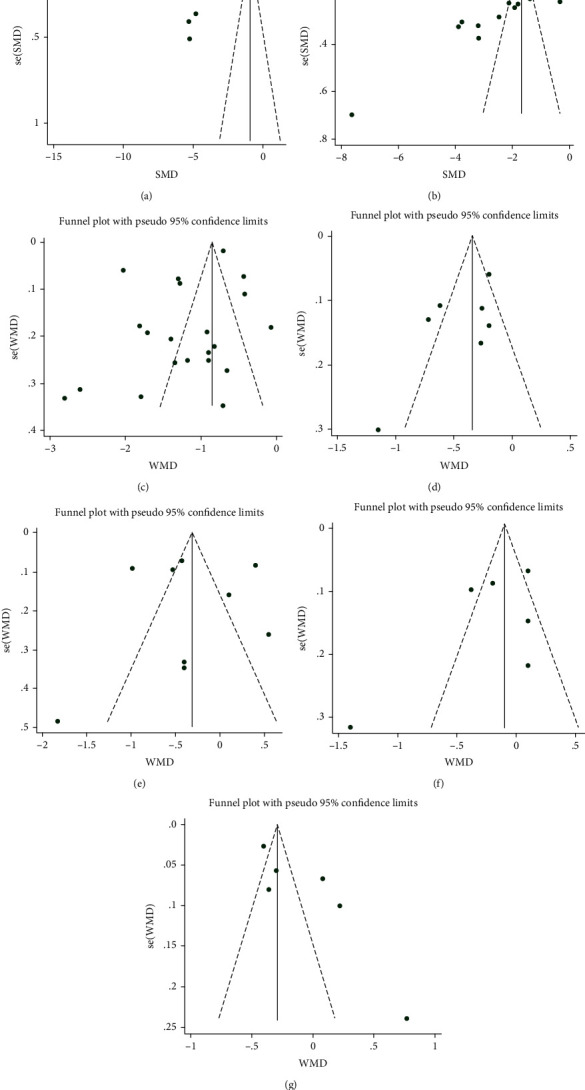
Funnel plots of intervention effect indexes ((a) FPG; (b) 2hPG; (c) HbA1c; (d) TC; (e) TG; (f) LDL-C; (g) HDL-C).

Funnel plots were used for detecting publication bias. In this analysis, the funnel plots appeared to be asymmetrical, suggesting a certain publication bias (Figure 9). The high heterogeneity and low-quality research methods included in the studies may be the reasons for any publication bias.

### 3.7. Grading of Quality of Evidence

Combined with relevant data, the certainty of evidence for each outcome was assessed using the GRADE approach, and a summary table was established using GRADEpro GDT software. Analysis indicated that the quality of evidence supporting the outcomes of the studies ranged from low to moderate due to inconsistency and publication bias (Table S4).

## 4. Discussion

To the best of our knowledge, this is the first SR of studies on HL intervention among people with diabetes in mainland China. Our findings indicate that HL intervention is effective in improving HL level and glycemic profile among people with diabetes, although improvements in lipid profile are less satisfactory.

### 4.1. Effectiveness of Intervention on HL

In general, it can be observed in our study that all patients who received the intervention showed a significant improvement in HL level. However, in terms of different dimensions, some patients only had an increase in HL knowledge level, and their calculation or application skills did not improve significantly [33, 43]. This may be related to the fact that interventions are mostly based on HE, which focuses excessively on knowledge indoctrination. It is a very complicated to achieve a qualitative change from accepting knowledge to using knowledge expertly [48]. As a result, it is possible that interventionists neglected to strengthen and validate patients' calculations or application skills during the intervention. Second, it is also possible that the duration of the intervention was too short to lead to such an outcome. Behavior change is a difficult process that requires continuous intervention and reinforcement of multiple factors over time, and the desired change may not be achieved through short-term training [56]. This phenomenon also suggests that future HL interventions should focus not only on the output of knowledge but also on the education and exercises in skills and application.

### 4.2. Effectiveness of Intervention on Glycolipid Metabolism

Previous studies have confirmed that the improvements in self-management are conducive to blood sugar control among people with diabetes [57]. Compared with conventional care, HL intervention can help patients distinguish their different diabetes care needs, thereby improving the relevance of care and their level of self-management. This is a positive factor that provides theoretical support for the self-management of people with diabetes. The results of our study also confirm this point: the level of self-management of patients was strengthened after HL intervention such that the control of FPG, 2hPG, and HbA1c was improved.

Disturbed glucose metabolism in diabetes predisposes to disturbed lipid metabolism, which is itself an independent risk factor for various chronic diabetes complications [58]. Consequently, previous studies have analyzed the effects of HL intervention on lipids in patients with diabetes. Some studies have demonstrated that HL interventions are effective in controlling the lipid profile of patients [15], while others have suggested that HL interventions were not beneficial for lipid control [43]. The structure of the diet, the mode of exercise, and the timing of the intervention may all contribute to this conflict. Diet and exercise are two healthy means of ameliorating blood lipid levels, but both approaches require a considerable amount of time to show an effect, with significant effects not necessarily occurring over a period of 3 or 6 months [59]. In the present study, HL intervention was not effective in controlling the lipid profile of people with diabetes.

### 4.3. Sources of Heterogeneity

In this meta-analysis, heterogeneity between studies was high, and neither the subgroup analysis of study design type nor the sensitivity analysis was able to identify sources of heterogeneity. Therefore, we further considered clinical aspects of heterogeneity. First, the interventions included differed between studies, with some focusing on dietary education while others adopted a teach-back approach to education, for example. Second, baseline HL levels differed among participants, which may potentially affect the effectiveness of the intervention. Third, the different methods of measuring indicator data could have an impact on outcomes. Fourth, the overall low assessment of methodological quality in the included studies may also lead to a high level of heterogeneity. Moreover, several studies showed that women and ethnic minorities were often underrepresented in clinical trials testing cardiovascular drugs, which may limit the generalizability of trial results to the entire population [60, 61]. This phenomenon reminds that the inequalities in enrollment of women and ethnic minorities in this study could lead to heterogeneity. As the studies included in this SR and meta-analysis did not provide data on the outcomes between men and women and ethnicity, we were unable to conduct further research on them. It is recommended that future HL intervention studies need to enhance the representativeness of clinical trials according to race and sex.

### 4.4. Innovations and Limitations

This study has a number of innovations. First, this study is the first to use an evidence-based medicine approach to systematically and comprehensively analyze multiple types of intervention for diabetes HL in mainland China and to quantitatively evaluate the improvement in glucose and lipid metabolism through meta-analysis to achieve a comprehensive evaluation of patient HL levels. Second, the majority of available studies adopted improvement in patient blood glucose as the evaluation index, with a lack of consideration for blood lipid evaluation. In addition to the analysis of blood glucose, this SR also conducted a combined analysis of changes in blood lipids after the intervention and more comprehensively evaluated the impact of HL intervention on biochemical indicators. Third, this study includes a comprehensive analysis and discussion of various HL assessment tools and elaborates on the improvement in patient HL after HL intervention.

The limitations of this study include the lack of a registration number in a SR registration platform (e.g., PROSPERO) for prereview, which may affect the transparency of the study, while the analysis of heterogeneity is not sufficiently comprehensive. Subgroup analysis was conducted only according to the type of study design, and subsequent analyses in the future could consider this from other perspectives, such as intervention method and intervention time.

## 5. Conclusion

The HL intervention enhanced the total health literacy level of people with diabetes and effectively improved glucose metabolism and TC levels, but did not significantly improve TG, LDL-C, or HDL-C. Subsequent study designs should include more scientific and rigorous high-quality intervention plans, including extending the intervention and follow-up time, considering the impact on patient behavior and skills, and clearing the long-term effect of HL. In addition, a set of diabetes HL evaluation index systems applicable to the population in mainland China should be established as soon as possible, so as to provide evaluation criteria and implementation references for the formulation of HL interventions for people with diabetes.

## Figures and Tables

**Table 1:** General characteristics of included studies.

First author, year (reference no.)	Study type	Sample size (*n*)	Man/woman (*n*)		Age (mean ± SD, year)		Measure		Intervention time
		T/C	T	C	T	C	T	C	
Bao JL, 2019 [33]	RCT	50/50	35/15	38/12	65.40 ± 3.78	66.60 ± 4.93	Teach-back health education	Routine health education	3 months
Cao YF, 2020 [23]	RCT	60/60	36/24	39/21	52.54 ± 3.89	51.42 ± 4.35	WeChat health education	Routine health education	3 months
Cheng L, 2017 [34]	RCT	121/121	93/28	86/35	56.13 ± 10.72	53.91 ± 13.01	Empowerment-based intervention	Health education	3 months
Chen YY, 2020 [22]	RCT	83/44	44/39	46/38	62.35 ± 11.21	60.80 ± 12.07	Teach-back method intervention	Traditional diabetes health education	3 months
Hao ML, 2019 [31]	RCT	44/44	24/20	26/18	63.35 ± 6.38	64.70 ± 7.05	Teach-back health education	Routine health education	Until discharge
Li CF, 2017 [43]	RCT	80/80	35/45	39/41	≥45^※^		Self-management and routine community health services	Routine community health services	3 months
Li L, 2020 [21]	RCT	42/41	22/20	18/23	59.60 ± 8.54	61.05 ± 9.09	Teach-back method combined with telephone follow-up intervention	Routine health service	6 months
Li L, 2020 [20]	RCT	40/40	21/19	23/17	68.70 ± 9.00	67.73 ± 10.39	Teach-back method combined with family involved	Routine health education	6 months
Liu QH, 2020 [19]	RCT	50/50	28/22	29/21	66.51 ± 1.24	67.21 ± 1.68	Five-full nursing modes as primary medical and nursing intervention	Traditional nursing care	Not reported
Liu QN, 2018 [40]	RCT	534/534	234/300	308/226	67.9 ± 10.1	67.8 ± 10.2	Health education	Traditional services	Not reported
Li YF, 2015 [52]	RCT	126/134	69/57	63/71	79.18 ± 8.81	79.07 ± 9.23	Teach-back and peer education	Peer education	4 months
Ma GZ, 2019 [29]	RCT	25/25	13/12	14/11	65.7 ± 4.51	66.4 ± 4.23	Five-full nursing modes as primary medical and nursing intervention	Routine nursing care	6 months
Peng Y, 2021 [15]	RCT	54/48	62/40		36~68^※^		Internet-based health education	Ordinary health education	6 months
Tu Y, 2018 [39]	RCT	54/54	26/28	25/29	68.91 ± 4.32	68.25 ± 4.35	Integrated intervention of family doctor based on health literacy	Routine community health services	1 year
Wang L, 2019 [24]	RCT	200/200	89/111	86/114	65.8 ± 9.7	63.8 ± 8.7	Health literacy education	Routine community health services	1 year
Wang Q, 2018 [38]	RCT	57/57	72/42		58.08 ± 6.30		Teach-back health education and routine nursing care	Routine nursing care	6 months
Wu P, 2019 [28]	RCT	55/55	29/26	30/25	28~77^※^	27~75^※^	Micro lecture of health literacy	Routine health education	Not reported
Xu YL, 2020 [10]	RCT	60/60	31/29	33/27	69.4 ± 7.2	68.5 ± 6.8	Community–hospital–family intervention	Routine nursing intervention	12 months
Yao XQ, 2019 [27]	RCT	191/188	130/61	126/62	65.7 ± 5.9	66.2 ± 6.1	Structured health education	Routine health care services	6 months
You HQ, 2020 [18]	RCT	35/35	20/15	19/16	75.6 ± 6.9	76.9 ± 7.0	Structured health education	Routine nursing care	Not reported
Yu F, 2020 [17]	RCT	60/60	25/35	21/39	73.02 ± 6.95	72.28 ± 6.97	Insulin interview tool intervention	Routine health education	3 months
Zhang JB, 2019 [26]	RCT	62/62	Not reported		Not reported		Personalized diabetes self-management	Routine health education	Not reported
Zhang N, 2018 [37]	RCT	60/60	34/26	32/28	42.6 ± 9.3	41.7 ± 8.9	Micro lecture of health literacy	Routine health education	Until discharge
Zhang RT, 2016 [47]	RCT	80/80	87/73		48 ± 10.3		Mental intervention and health knowledge guidance	Health knowledge guidance	6 months
Zhang XH, 2019 [25]	RCT	98/98	63/35	65/33	60.3 ± 6.7	61.0 ± 6.8	Health education and exercise intervention	Routine discharge guidance	3 months
Zhang Y, 2018 [36]	RCT	38/38	22/16	23/15	52.17 ± 3.64	52.12 ± 3.61	Comprehensive health literacy management	Routine community health services	Not reported
Zhao FY, 2018 [35]	RCT	45/45	25/20	24/21	51.8 ± 6.8	52.2 ± 6.9	Teach-back health education	Routine health education	6 weeks
Cheng LL, 2019 [32]	Non-RCT	50/50	25/25	25/25	57.3 ± 3.2	56.3 ± 3.4	Strengthen self-efficacy management and routine diabetes treatment	Routine diabetes treatment	Not reported
Li YJ, 2019 [30]	Non-RCT	40/40	21/19	21/19	56.0 ± 3.8	55.8 ± 3.9	Dietary intervention and routine community health services	Routine community health services	6 months
Ouyang HG, 2019 [9]	Non-RCT	791/1335	386/405	684/651	55.7 ± 10.5	54.8 ± 10.9	Personalized health education	Routine community health services	6 months
Pan JY, 2015 [51]	Non-RCT	38/38	20/18	20/18	57.1 ± 4.7	56.9 ± 4.6	Dietary intervention and routine community health services	Routine community health services	6 months
Qiao W, 2014 [53]	Non-RCT	100/100	Not reported		55.6 ± 21.7	55.5 ± 19.8	Traditional Chinese medicine health management and drug therapy	Drug therapy	1 year
Shi M, 2016 [45]	Non-RCT	60/60	32/28	26/34	57.87 ± 13.47	57.10 ± 11.38	Family involved in the whole health education	Single involved the whole health education	2 years
Wang N, 2021 [14]	Non-RCT	31/29	18/13	17/12	73.91 ± 4.86	73.85 ± 4.65	Teach-back method combined with video and health education	Teach-back method combined with health education	3 months
Yang FH, 2017 [41]	Non-RCT	555/502	219/336	225/277	63.60 ± 7.29	64.91 ± 6.63	Comprehensive health literacy management	Routine community health services	6 months
Zhang Y, 2016 [46]	Non-RCT	27/27	11/16	14/13	78.41 ± 4.53	78.0 ± 4.67	Micro lecture of health literacy	Routine nursing care	Not reported
Zhong Y, 2020 [16]	Non-RCT	33/33	19/14	18/15	70.12 ± 1.22	70.11 ± 1.21	Health promotion management	Routine outpatient management	3 months
Chen GM, 2013 [54]	CBA	215/—	114/104		56.0 ± 12.2		Community nursing intervention	—	1 year
Gao YY, 2017 [44]	CBA	110/—	58/52		62.3 ± 11.1		Diabetes self-management education	—	7 months
Hu GS, 2016 [49]	CBA	210/—	90/120		45~75^※^		Health literacy education	—	6 months
Li SF, 2010 [55]	CBA	121/—	64/57		66.0 ± 19.1		A nurse-led clinic on health literacy	—	1 month
Liu XM, 2016 [48]	CBA	68/—	31/37		48~75^※^		Health education	—	8 months
Su QX, 2017 [42]	CBA	286/—	152/134		69.08 ± 3.25		Comprehensive intervention measures of health literacy	—	Not reported
Wen XQ, 2015 [50]	CBA	413/—	198/215		67.4 ± 10.5		Team-based individualized health management intervention	—	Not reported

CBA: controlled before-and-after trial; non-RCT: nonrandomized controlled trial; RCT: randomized controlled trial; T means intervention group; C means control group; SD: standard deviation; ^※^age range was reported if mean ± standard deviation was not available.

**Table 2:** Quality assessment of included studies.

First author, year (reference no.)	Type	①	②	③	④	⑤	⑥	⑦	⑧	⑨	Score
Bao JL, 2019 [33]	RCT	Yes	Unclear	Yes	Yes	Yes	Yes	Unclear	Yes	Yes	7
Cao YF, 2020 [23]	RCT	Yes	Unclear	Yes	Yes	Yes	Yes	Unclear	Yes	Yes	7
Cheng L, 2017 [34]	RCT	Yes	Yes	Yes	Yes	Yes	Yes	Unclear	Yes	Yes	8
Chen YY, 2020 [22]	RCT	Yes	Unclear	Yes	Yes	Yes	Yes	Unclear	Yes	Yes	7
Hao ML, 2019 [31]	RCT	Yes	Unclear	Yes	Yes	Yes	Yes	Unclear	Yes	Yes	7
Li CF, 2017 [43]	RCT	Unclear	Unclear	Yes	Yes	Yes	Yes	Unclear	Yes	Yes	6
Li L, 2020 [21]	RCT	Yes	Unclear	Yes	Yes	Yes	Yes	Unclear	Yes	Yes	7
Li L, 2020 [20]	RCT	Yes	Unclear	Yes	Yes	Yes	Yes	Unclear	Yes	Yes	7
Liu QH, 2020 [19]	RCT	No	Unclear	Yes	Yes	Yes	Yes	Unclear	Yes	Yes	6
Liu QN, 2018 [40]	RCT	Unclear	Unclear	Yes	Yes	Yes	Yes	Unclear	Yes	Yes	6
Li YF, 2015 [52]	RCT	Yes	Unclear	Yes	Yes	Yes	Yes	Unclear	Yes	Yes	7
Ma GZ, 2019 [29]	RCT	Yes	Unclear	Yes	Yes	Yes	Yes	Unclear	Yes	Yes	7
Peng Y, 2021 [15]	RCT	Yes	Unclear	Yes	Yes	Yes	Yes	Unclear	Yes	Yes	7
Tu Y, 2018 [39]	RCT	Yes	Unclear	Yes	Yes	Yes	Yes	Unclear	Yes	Yes	7
Wang L, 2019 [24]	RCT	Yes	Yes	Yes	No	Yes	Yes	Unclear	Yes	Yes	7
Wang Q, 2018 [38]	RCT	Unclear	Unclear	Yes	Yes	Yes	Yes	Unclear	Yes	Yes	6
Wu P, 2019 [28]	RCT	Unclear	Unclear	Yes	Yes	Yes	Yes	Unclear	Yes	Yes	6
Xu YL, 2020 [10]	RCT	Yes	Unclear	Yes	Yes	Yes	Yes	Unclear	Yes	Yes	7
Yao XQ, 2019 [27]	RCT	Yes	Unclear	Yes	Yes	Yes	Yes	Unclear	Yes	Yes	7
You HQ, 2020 [18]	RCT	Yes	Unclear	Yes	Yes	Yes	Yes	Unclear	Yes	Yes	7
Yu F, 2020 [17]	RCT	Yes	Unclear	Yes	Yes	Yes	Yes	Unclear	Yes	Yes	7
Zhang JB, 2019 [26]	RCT	Unclear	Unclear	Yes	Yes	Yes	Yes	Unclear	Yes	Yes	6
Zhang N, 2018 [37]	RCT	Yes	Unclear	Yes	Yes	Yes	Yes	Unclear	Yes	Yes	7
Zhang RT, 2016 [47]	RCT	Unclear	Unclear	Unclear	Yes	Yes	Yes	Unclear	Yes	Yes	5
Zhang XH, 2019 [25]	RCT	Yes	Unclear	Yes	Yes	Yes	Yes	Unclear	Yes	Yes	7
Zhang Y, 2018 [36]	RCT	No	Unclear	Yes	Yes	Yes	Yes	Unclear	Yes	Yes	6
Zhao FY, 2018 [35]	RCT	Yes	Unclear	Yes	Yes	Yes	Yes	Unclear	Yes	Yes	7
Cheng LL, 2019 [32]	Non-RCT	Unclear	Unclear	Yes	Yes	Yes	Yes	Unclear	Yes	Yes	6
Li YJ, 2019 [30]	Non-RCT	No	No	Yes	Yes	Yes	Yes	Unclear	Yes	Yes	6
Ouyang HG, 2019 [9]	Non-RCT	No	No	Yes	Yes	Yes	Yes	Unclear	Yes	Yes	6
Pan JY, 2015 [51]	Non-RCT	No	No	Yes	Yes	Yes	Yes	Unclear	Yes	Yes	6
Qiao W, 2014 [53]	Non-RCT	No	No	Yes	Yes	Yes	Yes	Unclear	Yes	Yes	6
Shi M, 2016 [45]	Non-RCT	No	No	Yes	Yes	Yes	Yes	Unclear	Yes	Yes	6
Wang N, 2021 [14]	Non-RCT	No	No	Yes	Yes	Yes	Yes	Unclear	Yes	Yes	6
Yang FH, 2017 [41]	Non-RCT	No	No	Yes	Yes	Yes	Yes	Unclear	Yes	Yes	6
Zhang Y, 2016 [46]	Non-RCT	No	No	Yes	Yes	Yes	Yes	Unclear	Yes	Yes	6
Zhong Y, 2020 [16]	Non-RCT	No	No	Yes	Yes	Yes	Yes	Unclear	Yes	Yes	6
Chen GM, 2013 [54]	CBA	No	No	Yes	Yes	Yes	Yes	Unclear	Yes	Yes	6
Gao YY, 2017 [44]	CBA	No	No	Yes	Yes	Yes	Yes	Unclear	Yes	Yes	6
Hu GS, 2016 [49]	CBA	No	No	Yes	Yes	Yes	Yes	Unclear	Yes	Yes	6
Li SF, 2010 [55]	CBA	No	No	Yes	Yes	Yes	Yes	Unclear	Yes	Yes	6
Liu XM, 2016 [48]	CBA	No	No	Yes	Yes	Yes	Yes	Unclear	Yes	Yes	6
Su QX, 2017 [42]	CBA	No	No	Yes	Yes	Yes	Yes	Unclear	Yes	Yes	6
Wen XQ, 2015 [50]	CBA	No	No	Yes	Yes	No	Yes	Unclear	Yes	Yes	5

①: random sequence generation; ②: allocation concealment; ③: baseline outcome measurements similar; ④: baseline characteristics similar; ⑤: incomplete outcome data; ⑥: knowledge of the allocated interventions adequately prevented during the study; ⑦: protection against contamination; ⑧: selective outcome reporting; ⑨: other risks of bias; CBA: controlled before-and-after trials; non-RCT: nonrandomized controlled trials; RCT: randomized controlled trials.

## Supplementary Materials

Supplementary Materials Table S1: PRISMA checklists. Table S2: search strategy. Table S3: contents of health literacy assessment tools. Table S4: summary of finding table. Figure S1: usage distribution of health literacy assessment tools. Figure S2: forest plot of FPG by subgroup analysis. Figure S3: forest plot of 2hPG by subgroup analysis. Figure S4: forest plot of HbA1c by subgroup analysis. Figure S5: forest plot of TC by subgroup analysis. Figure S6: forest plot of TG by subgroup analysis. Figure S7: forest plot of LDL-C by subgroup analysis. Figure S8: forest plot of HDL-C by subgroup analysis. Figure S9: sensitivity analysis of intervention effect indexes (A: FPG; B: 2hPG; C: HbA1c; D: TC; E: TG; F: LDL-C; G: HDL-C).
