# Supplementary material for: Effects of Health Literacy Intervention on Health Literacy Level and Glucolipid Metabolism of Diabetic Patients in Mainland China: A Systematic Review and Meta-Analysis
Source: J Diabetes Res. 2021 Dec 30;2021:1503446. doi: 10.1155/2021/1503446 (PMC8739182; doi:10.1155/2021/1503446)
Supplement: Supplementary Materials — Table S1: PRISMA checklists. Table S2: search strategy. Table S3: contents of health literacy assessment tools. Table S4: summary of finding table. Figure S1: usage distribution of health literacy assessment tools. Figure S2: forest plot of FPG by subgroup analysis. Figure S3: forest plot of 2hPG by subgroup analysis. Figure S4: forest plot of HbA1c by subgroup analysis. Figure S5: forest plot of TC by subgroup analysis. Figure S6: forest plot of TG by subgroup analysis. Figure S7: forest plot of LDL-C by subgroup analysis. Figure S8: forest plot of HDL-C by subgroup analysis. Figure S9: sensitivity analysis of intervention effect indexes (A: FPG; B: 2hPG; C: HbA1c; D: TC; E: TG; F: LDL-C; G: HDL-C). [file 1503446.f1.zip › Supplementary tables.docx]

**Table S1：PRISMA 2020 checklists.**

| **Section and Topic** | **Item #** | **Checklist item** | **Location where item is reported** |
| --- | --- | --- | --- |
| **TITLE** | | |  |
| Title | 1 | Identify the report as a systematic review. | Line 2～3 |
| **ABSTRACT** | | |  |
| Abstract | 2 | See the PRISMA 2020 for Abstracts checklist. | Line 13～32 |
| **INTRODUCTION** | | |  |
| Rationale | 3 | Describe the rationale for the review in the context of existing knowledge. | Line 37～57 |
| Objectives | 4 | Provide an explicit statement of the objective(s) or question(s) the review addresses. | Line 58～62 |
| **METHODS** | | |  |
| Eligibility criteria | 5 | Specify the inclusion and exclusion criteria for the review and how studies were grouped for the syntheses. | Line 81～86 |
| Information sources | 6 | Specify all databases, registers, websites, organisations, reference lists and other sources searched or consulted to identify studies. Specify the date when each source was last searched or consulted. | Line 70～75 |
| Search strategy | 7 | Present the full search strategies for all databases, registers and websites, including any filters and limits used. | Table S2 |
| Selection process | 8 | Specify the methods used to decide whether a study met the inclusion criteria of the review, including how many reviewers screened each record and each report retrieved, whether they worked independently, and if applicable, details of automation tools used in the process. | Line 78～80 |
| Data collection process | 9 | Specify the methods used to collect data from reports, including how many reviewers collected data from each report, whether they worked independently, any processes for obtaining or confirming data from study investigators, and if applicable, details of automation tools used in the process. | Line 78～80 |
| Data items | 10a | List and define all outcomes for which data were sought. Specify whether all results that were compatible with each outcome domain in each study were sought (e.g. for all measures, time points, analyses), and if not, the methods used to decide which results to collect. | Line 89～91 |
|  | 10b | List and define all other variables for which data were sought (e.g. participant and intervention characteristics, funding sources). Describe any assumptions made about any missing or unclear information. | Line 89～91 |
| Study risk of bias assessment | 11 | Specify the methods used to assess risk of bias in the included studies, including details of the tool(s) used, how many reviewers assessed each study and whether they worked independently, and if applicable, details of automation tools used in the process. | Line 92～98 |
| Effect measures | 12 | Specify for each outcome the effect measure(s) (e.g. risk ratio, mean difference) used in the synthesis or presentation of results. | Line 104～105 |
| Synthesis methods | 13a | Describe the processes used to decide which studies were eligible for each synthesis (e.g. tabulating the study intervention characteristics and comparing against the planned groups for each synthesis (item #5)). | —— |
|  | 13b | Describe any methods required to prepare the data for presentation or synthesis, such as handling of missing summary statistics, or data conversions. | —— |
|  | 13c | Describe any methods used to tabulate or visually display results of individual studies and syntheses. | —— |
|  | 13d | Describe any methods used to synthesize results and provide a rationale for the choice(s). If meta-analysis was performed, describe the model(s), method(s) to identify the presence and extent of statistical heterogeneity, and software package(s) used. | Line 101～104 |
|  | 13e | Describe any methods used to explore possible causes of heterogeneity among study results (e.g. subgroup analysis, meta-regression). | —— |
|  | 13f | Describe any sensitivity analyses conducted to assess robustness of the synthesized results. | Line 106～107 |
| Reporting bias assessment | 14 | Describe any methods used to assess risk of bias due to missing results in a synthesis (arising from reporting biases). | Line 107 |
| Certainty assessment | 15 | Describe any methods used to assess certainty (or confidence) in the body of evidence for an outcome. | —— |
| **RESULTS** | | |  |
| Study selection | 16a | Describe the results of the search and selection process, from the number of records identified in the search to the number of studies included in the review, ideally using a flow diagram. | Figure 1 |
|  | 16b | Cite studies that might appear to meet the inclusion criteria, but which were excluded, and explain why they were excluded. | Line 111～114 |
| Study characteristics | 17 | Cite each included study and present its characteristics. | Table 1 |
| Risk of bias in studies | 18 | Present assessments of risk of bias for each included study. | Table 2 |
| Results of individual studies | 19 | For all outcomes, present, for each study: (a) summary statistics for each group (where appropriate) and (b) an effect estimate and its precision (e.g. confidence/credible interval), ideally using structured tables or plots. | Line 141～158 |
| Results of syntheses | 20a | For each synthesis, briefly summarise the characteristics and risk of bias among contributing studies. | —— |
|  | 20b | Present results of all statistical syntheses conducted. If meta-analysis was done, present for each the summary estimate and its precision (e.g. confidence/credible interval) and measures of statistical heterogeneity. If comparing groups, describe the direction of the effect. | Line 169～231 |
|  | 20c | Present results of all investigations of possible causes of heterogeneity among study results. | —— |
|  | 20d | Present results of all sensitivity analyses conducted to assess the robustness of the synthesized results. | Figure S9 |
| Reporting biases | 21 | Present assessments of risk of bias due to missing results (arising from reporting biases) for each synthesis assessed. | Line 239～241, Figure 9 |
| Certainty of evidence | 22 | Present assessments of certainty (or confidence) in the body of evidence for each outcome assessed. | Line 246～249, Table S4 |
| **DISCUSSION** | | |  |
| Discussion | 23a | Provide a general interpretation of the results in the context of other evidence. | Line 252～284 |
|  | 23b | Discuss any limitations of the evidence included in the review. | Line 287～293 |
|  | 23c | Discuss any limitations of the review processes used. | Line 305～309 |
|  | 23d | Discuss implications of the results for practice, policy, and future research. | Line 312～318 |
| **OTHER INFORMATION** | | |  |
| Registration and protocol | 24a | Provide registration information for the review, including register name and registration number, or state that the review was not registered. | Line 305～307 |
|  | 24b | Indicate where the review protocol can be accessed, or state that a protocol was not prepared. | —— |
|  | 24c | Describe and explain any amendments to information provided at registration or in the protocol. | —— |
| Support | 25 | Describe sources of financial or non-financial support for the review, and the role of the funders or sponsors in the review. | Line 330～331 |
| Competing interests | 26 | Declare any competing interests of review authors. | Line 328 |
| Availability of data, code and other materials | 27 | Report which of the following are publicly available and where they can be found: template data collection forms; data extracted from included studies; data used for all analyses; analytic code; any other materials used in the review. | Line 320 |

**Table S2:** **Search strategy.**

| **PubMed** | |
| --- | --- |
| #1 | health literacy[title/abstract] |
| #2 | diabetes mellitus[title/abstract] OR dm[title/abstract] OR diabetes[title/abstract] OR diabetic mellitus[title/abstract] OR diabetic[title/abstract] OR mellitus[title/abstract] |
| #3 | #1 AND #2 |
| **SCIE of Web of Science** | |
| #1 | health literacy[topic] |
| #2 | diabetes mellitus[topic] OR dm[topic] OR diabetes[topic] OR diabetic mellitus[topic] OR diabetic[topic] OR mellitus[topic] |
| #3 | #1 AND #2 |
| **EMbase** | |
| #1 | diabetes mellitus/ |
| #2 | health literacy.ti. or health literacy.kw. or health literacy.ab. |
| #3 | (diabetes mellitus or diabetes or diabetic or DM).ti. or diabetes mellitus.kw. or diabetes.kw. or diabetic.kw. or DM.kw. or diabetes mellitus.ab. or diabetes.ab. or diabetic.ab. or DM.ab. |
| #4 | health literacy/ |
| #5 | #1 or #3 |
| #6 | #2 or #4 |
| #7 | #5 and #6 |
| **CNKI and Wanfang database** | |
| #1 | 糖尿病[topic] |
| #2 | 健康素养[topic] |
| #3 | #1 AND #2 |
| **CQVIP** | |
| #1 | 糖尿病[ti/kw] |
| #2 | 健康素养[ti/kw] |
| #3 | #1 AND #2 |
| #4 | 糖尿病[ab] |
| #5 | 健康素养[ab] |
| #6 | #4 AND #5 |
| #7 | #3 OR #6 |

**Table S3：****Contents of health literacy assessment tools.**

| Health literacy assessment tool | Content |
| --- | --- |
| Health Literacy Management Scale （HeLMS） | ①Understanding health information; ②Communication with health professionals;  ③Patient attitudes towards their health; ④Socioeconomic considerations. |
| Diabetes Health Literacy Assessment Tool designed by Miyong Kim | ①Numeracy skill; ②Reading skill; ③Comprehension skill. |
| Chinese Public Diabetes Prevention and Treatment Literacy Questionnaire | ①Basic knowledge of diabetes mellitus; ②Diabetes prevention behavior;  ③Access to and utilization of diabetes information. |
| Chinese Citizens Health Literacy Questionnaire | ①Basic knowledge and concept of health; ②Healthy lifestyle and behavior;  ③Health skills |
| Health Literacy Questionnaire for Diabetic Patients | ①Sports knowledge; ②Basic knowledge of prevention and control;  ③Knowledge and behavior of dietary therapy; ④Psychological and medical knowledge. |
| Self-designed questionnaire |  |
| Diabetes Health Literacy Questionnaire | ①Health knowledge; ②Health belief; ③Healthy behavior；④Health skills. |
| Health Literacy Questionnaire for Community Type 2 Diabetic Patients | ①Basic knowledge of prevention and control; ②Sports knowledge；③Knowledge of medicine and psychology; ④Knowledge of dietary therapy; ⑤Prevention behavior. |
| Simple Health Literacy Assessment Form for Elderly Diabetes | ①Memory; ②Understanding；③Analysis；④Applications. |
| Diabetes Knowledge, Skills and Behavior Questionnaire | ①Drug therapy for diabetes；②Diet；③Sports；④Disease Control；  ⑤Self-monitoring; ⑥Skills and patient behaviour. |
| Health Literacy Assessment Tool for Diabetic Patients | ①Health concept；②Communicable disease prevention；③Health behavior；  ④Basic medical；⑤Safety. |

**Table S4：****Summary of finding table.**

| **Certainty assessment** | | | | | | | **№ of patients** | | **Effect** | **Certainty** | **Importance** |
| --- | --- | --- | --- | --- | --- | --- | --- | --- | --- | --- | --- |
| **№ of studies** | **Study design** | **Risk of bias** | **Inconsistency** | **Indirectness** | **Imprecision** | **Other considerations** | **health literacy intervention** | **usual care** | **Absolute (95% CI)** |  |  |
| **FPG** | | | | | | | | | | | |
| 18 | randomised trials | not serious | serious^a^ | not serious | not serious | publication bias strongly suspected^b^ | 1225（1215） | 1219（1104） | **SMD**=-1.81, **95%CI=** (-2.35 to -1.27) | ⨁⨁◯◯ Low | CRITICAL |
| **2hPG** | | | | | | | | | | | |
| 15 | randomised trials | not serious | serious^a^ | not serious | not serious | publication bias strongly suspected^b^ | 885（884） | 879（876） | **SMD**=-2.29, **95%CI=** (-2.86 to-1.72) | ⨁⨁◯◯ Low | CRITICAL |
| **HbA1c** | | | | | | | | | | | |
| 16 | randomised trials | not serious | serious^a^ | not serious | not serious | publication bias strongly suspected^c^ | 1342（1332） | 1336（1321） | **WMD**=-1.13, **95%CI=** (-1.37 to -0.89) | ⨁⨁◯◯ Low | CRITICAL |
| **TC** | | | | | | | | | | | |
| 6 | randomised trials | not serious | serious^a^ | not serious | not serious | none | 505（496） | 499（487） | **WMD**=-0.49, **95%CI=** (-0.71 to -0.26) | ⨁⨁⨁◯ Moderate | IMPORTANT |
| **TG** | | | | | | | | | | | |
| 6 | randomised trials | not serious | serious^a^ | not serious | not serious | none | 505（496） | 499（487） | **WMD**=-0.44, **95%CI=** (-0.82 to- 0.07) | ⨁⨁⨁◯ Moderate | IMPORTANT |
| **LDL-C** | | | | | | | | | | | |
| 5 | randomised trials | not serious | serious^a^ | not serious | not serious | none | 448（439） | 442（430） | **WMD**=-0.28, **95%CI=** (-0.58 to 0.03) | ⨁⨁⨁◯ Moderate | IMPORTANT |
| **HDL_C** | | | | | | | | | | | |
| 5 | randomised trials | not serious | serious^a^ | not serious | not serious | none | 448（439） | 442（430） | **WMD**=0.03, **95%CI=** (-0.25 to 0.3) | ⨁⨁⨁◯ Moderate | IMPORTANT |

**Explanations**

1. I²＞50%, p≤0.1. (**FPG:** I-squared=97.0%, P=0.000; **2hPG:** I-squared=95.6%, P=0.000; **HbA1c:** I-squared=93.6%, P=0.000; **TC:** I-squared=74.3%, P=0.002; **TG:** I-squared=92.6%, P=0.000; **LDL-C:** I-squared=83.0%, P=0.000; **HDL_C:** I-squared=92.5%, P=0.000.).

b. Begg’s Test, P<0.05; Egger’s Test, P<0.05. (**FPG:** Begg’s Test, P=0.000; Egger’s Test, P=0.000. **2hPG:** Begg’s Test, P=0.008; Egger’s Test, P=0.002.).

c. Begg’s Test, P=0.192; Egger’s Test, P=0.049. (Egger’s Test is recommended to be used even though Begg’s Test P>0.1 indicates that there is no publication bias.).
