# Supplementary figures and images for: Effects of Health Literacy Intervention on Health Literacy Level and Glucolipid Metabolism of Diabetic Patients in Mainland China: A Systematic Review and Meta-Analysis
Source: J Diabetes Res. 2021 Dec 30;2021:1503446. doi: 10.1155/2021/1503446 (PMC8739182; doi:10.1155/2021/1503446)

The name of HL assessment tools

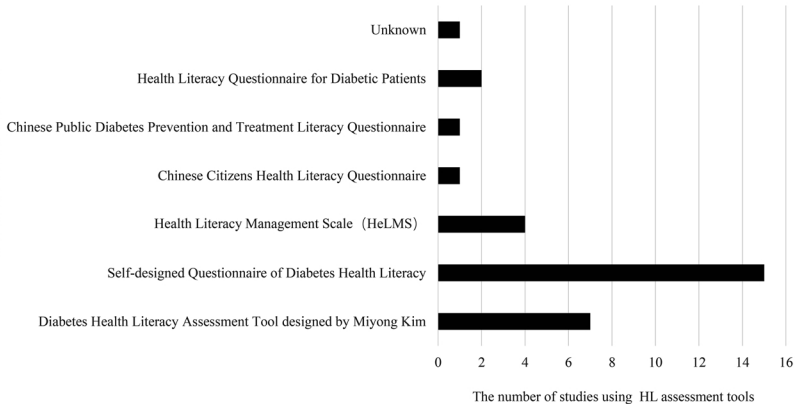

Supplement: Supplementary Materials — Table S1: PRISMA checklists. Table S2: search strategy. Table S3: contents of health literacy assessment tools. Table S4: summary of finding table. Figure S1: usage distribution of health literacy assessment tools. Figure S2: forest plot of FPG by subgroup analysis. Figure S3: forest plot of 2hPG by subgroup analysis. Figure S4: forest plot of HbA1c by subgroup analysis. Figure S5: forest plot of TC by subgroup analysis. Figure S6: forest plot of TG by subgroup analysis. Figure S7: forest plot of LDL-C by subgroup analysis. Figure S8: forest plot of HDL-C by subgroup analysis. Figure S9: sensitivity analysis of intervention effect indexes (A: FPG; B: 2hPG; C: HbA1c; D: TC; E: TG; F: LDL-C; G: HDL-C). [file 1503446.f1.zip › Figure S1-Usage distribution of health literacy assessment tools.pdf]

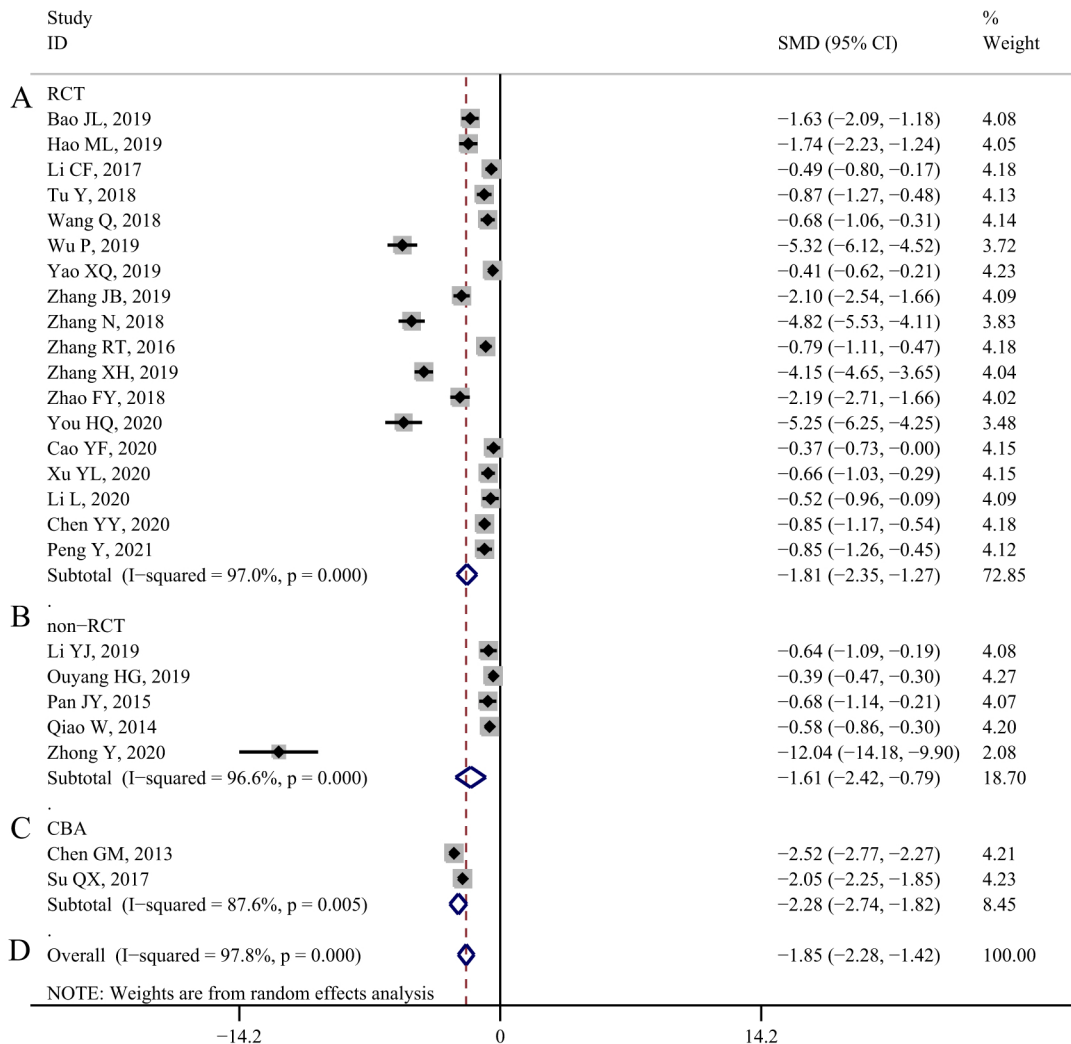

Supplement: Supplementary Materials — Table S1: PRISMA checklists. Table S2: search strategy. Table S3: contents of health literacy assessment tools. Table S4: summary of finding table. Figure S1: usage distribution of health literacy assessment tools. Figure S2: forest plot of FPG by subgroup analysis. Figure S3: forest plot of 2hPG by subgroup analysis. Figure S4: forest plot of HbA1c by subgroup analysis. Figure S5: forest plot of TC by subgroup analysis. Figure S6: forest plot of TG by subgroup analysis. Figure S7: forest plot of LDL-C by subgroup analysis. Figure S8: forest plot of HDL-C by subgroup analysis. Figure S9: sensitivity analysis of intervention effect indexes (A: FPG; B: 2hPG; C: HbA1c; D: TC; E: TG; F: LDL-C; G: HDL-C). [file 1503446.f1.zip › Figure S2-Forest plot of FPG by subgroup analysis.pdf]

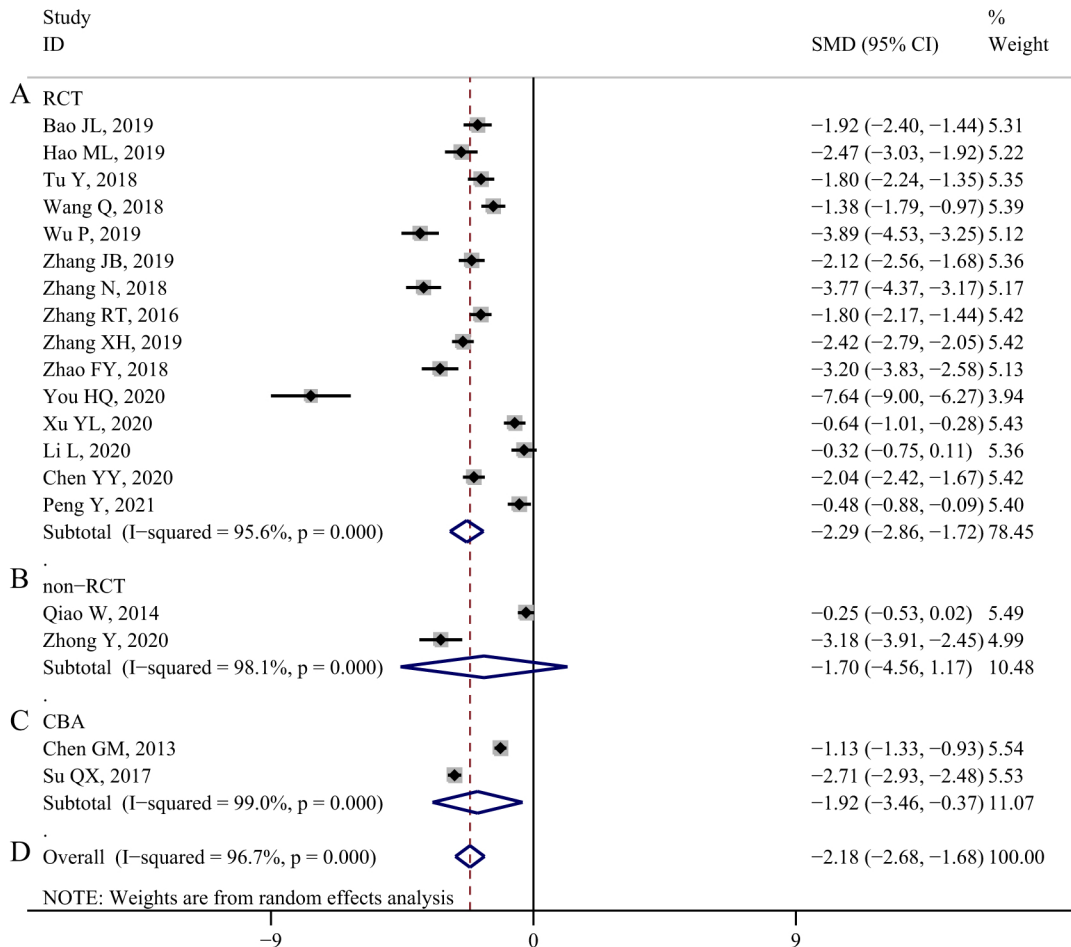

Supplement: Supplementary Materials — Table S1: PRISMA checklists. Table S2: search strategy. Table S3: contents of health literacy assessment tools. Table S4: summary of finding table. Figure S1: usage distribution of health literacy assessment tools. Figure S2: forest plot of FPG by subgroup analysis. Figure S3: forest plot of 2hPG by subgroup analysis. Figure S4: forest plot of HbA1c by subgroup analysis. Figure S5: forest plot of TC by subgroup analysis. Figure S6: forest plot of TG by subgroup analysis. Figure S7: forest plot of LDL-C by subgroup analysis. Figure S8: forest plot of HDL-C by subgroup analysis. Figure S9: sensitivity analysis of intervention effect indexes (A: FPG; B: 2hPG; C: HbA1c; D: TC; E: TG; F: LDL-C; G: HDL-C). [file 1503446.f1.zip › Figure S3-Forest plot of 2hPG by subgroup analysis.pdf]

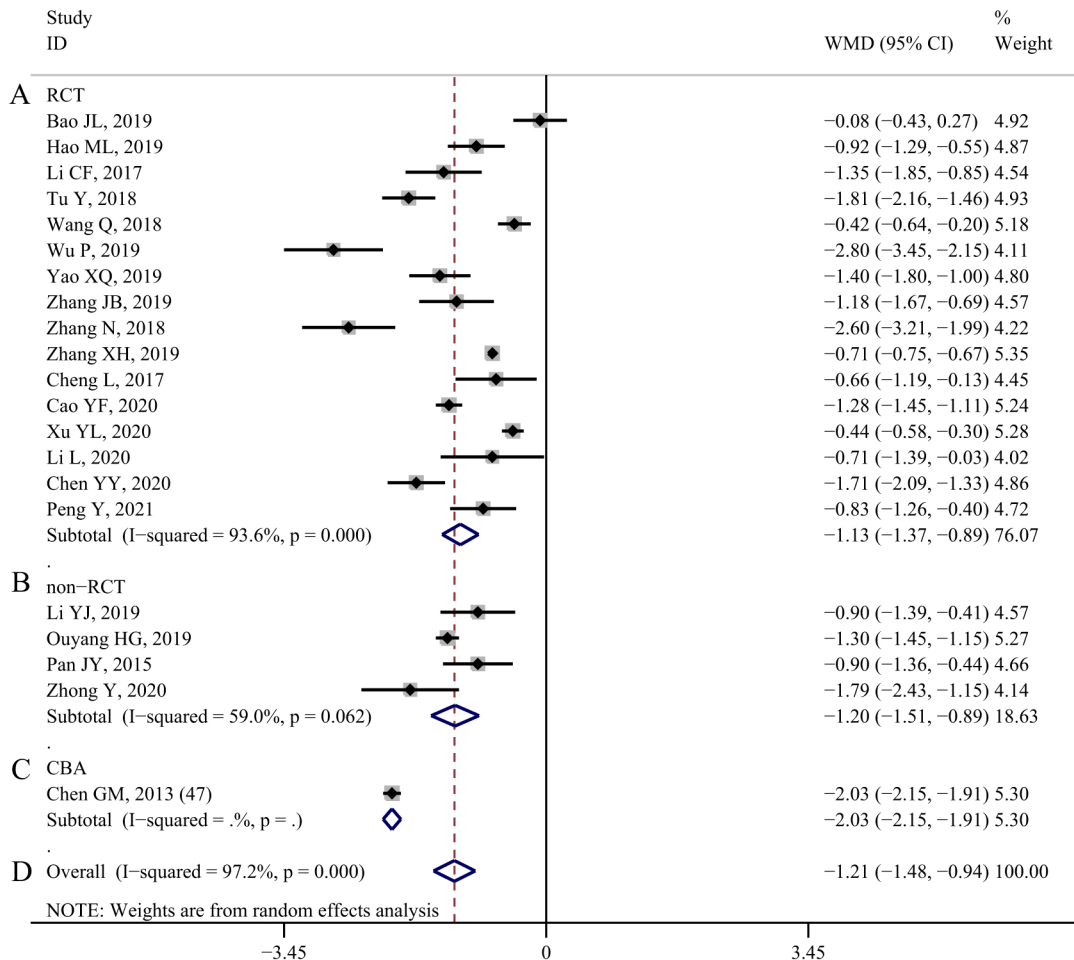

Supplement: Supplementary Materials — Table S1: PRISMA checklists. Table S2: search strategy. Table S3: contents of health literacy assessment tools. Table S4: summary of finding table. Figure S1: usage distribution of health literacy assessment tools. Figure S2: forest plot of FPG by subgroup analysis. Figure S3: forest plot of 2hPG by subgroup analysis. Figure S4: forest plot of HbA1c by subgroup analysis. Figure S5: forest plot of TC by subgroup analysis. Figure S6: forest plot of TG by subgroup analysis. Figure S7: forest plot of LDL-C by subgroup analysis. Figure S8: forest plot of HDL-C by subgroup analysis. Figure S9: sensitivity analysis of intervention effect indexes (A: FPG; B: 2hPG; C: HbA1c; D: TC; E: TG; F: LDL-C; G: HDL-C). [file 1503446.f1.zip › Figure S4-Forest plot of HbA1c by subgroup analysis.pdf]

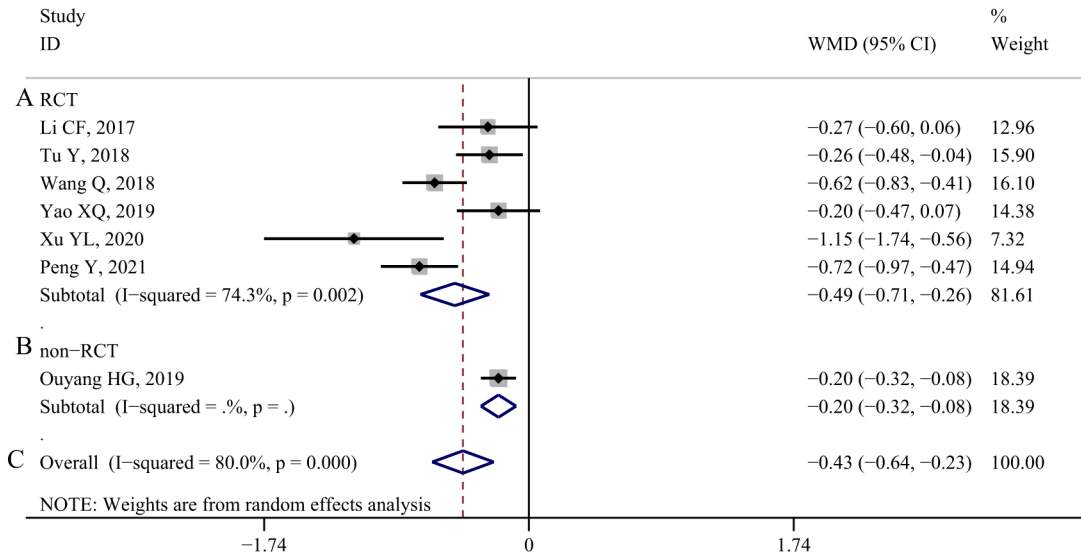

Supplement: Supplementary Materials — Table S1: PRISMA checklists. Table S2: search strategy. Table S3: contents of health literacy assessment tools. Table S4: summary of finding table. Figure S1: usage distribution of health literacy assessment tools. Figure S2: forest plot of FPG by subgroup analysis. Figure S3: forest plot of 2hPG by subgroup analysis. Figure S4: forest plot of HbA1c by subgroup analysis. Figure S5: forest plot of TC by subgroup analysis. Figure S6: forest plot of TG by subgroup analysis. Figure S7: forest plot of LDL-C by subgroup analysis. Figure S8: forest plot of HDL-C by subgroup analysis. Figure S9: sensitivity analysis of intervention effect indexes (A: FPG; B: 2hPG; C: HbA1c; D: TC; E: TG; F: LDL-C; G: HDL-C). [file 1503446.f1.zip › Figure S5-Forest plot of TC by subgroup analysis.pdf]

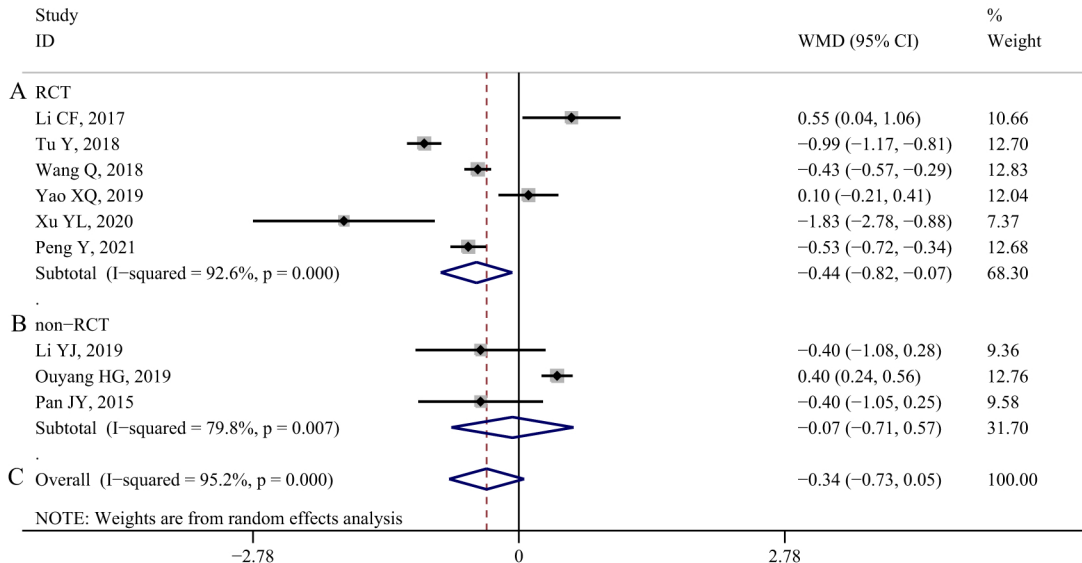

Supplement: Supplementary Materials — Table S1: PRISMA checklists. Table S2: search strategy. Table S3: contents of health literacy assessment tools. Table S4: summary of finding table. Figure S1: usage distribution of health literacy assessment tools. Figure S2: forest plot of FPG by subgroup analysis. Figure S3: forest plot of 2hPG by subgroup analysis. Figure S4: forest plot of HbA1c by subgroup analysis. Figure S5: forest plot of TC by subgroup analysis. Figure S6: forest plot of TG by subgroup analysis. Figure S7: forest plot of LDL-C by subgroup analysis. Figure S8: forest plot of HDL-C by subgroup analysis. Figure S9: sensitivity analysis of intervention effect indexes (A: FPG; B: 2hPG; C: HbA1c; D: TC; E: TG; F: LDL-C; G: HDL-C). [file 1503446.f1.zip › Figure S6-Forest plot of TG by subgroup analysis.pdf]

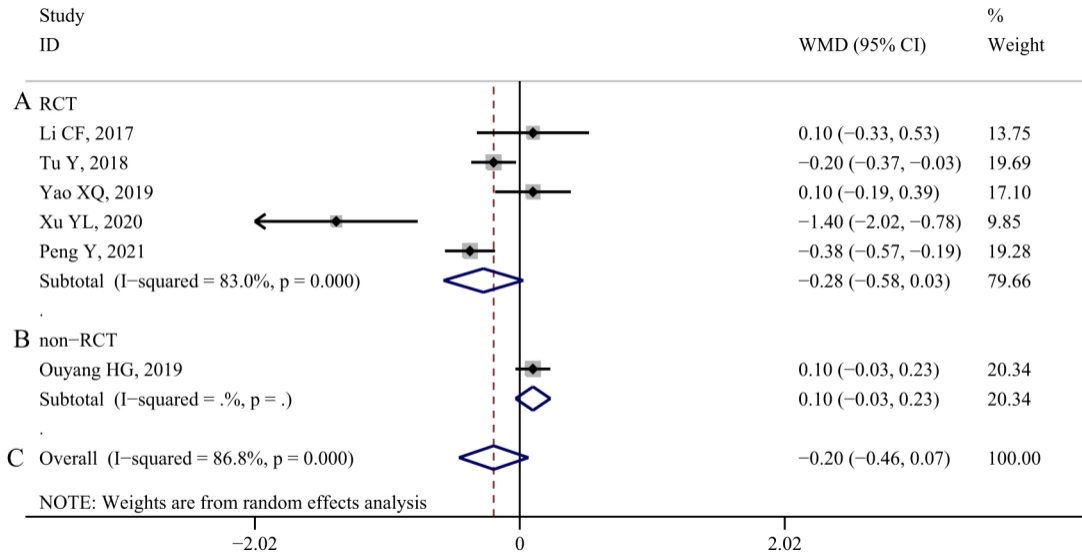

Supplement: Supplementary Materials — Table S1: PRISMA checklists. Table S2: search strategy. Table S3: contents of health literacy assessment tools. Table S4: summary of finding table. Figure S1: usage distribution of health literacy assessment tools. Figure S2: forest plot of FPG by subgroup analysis. Figure S3: forest plot of 2hPG by subgroup analysis. Figure S4: forest plot of HbA1c by subgroup analysis. Figure S5: forest plot of TC by subgroup analysis. Figure S6: forest plot of TG by subgroup analysis. Figure S7: forest plot of LDL-C by subgroup analysis. Figure S8: forest plot of HDL-C by subgroup analysis. Figure S9: sensitivity analysis of intervention effect indexes (A: FPG; B: 2hPG; C: HbA1c; D: TC; E: TG; F: LDL-C; G: HDL-C). [file 1503446.f1.zip › Figure S7-Forest plot of LDL-C by subgroup analysis.pdf]

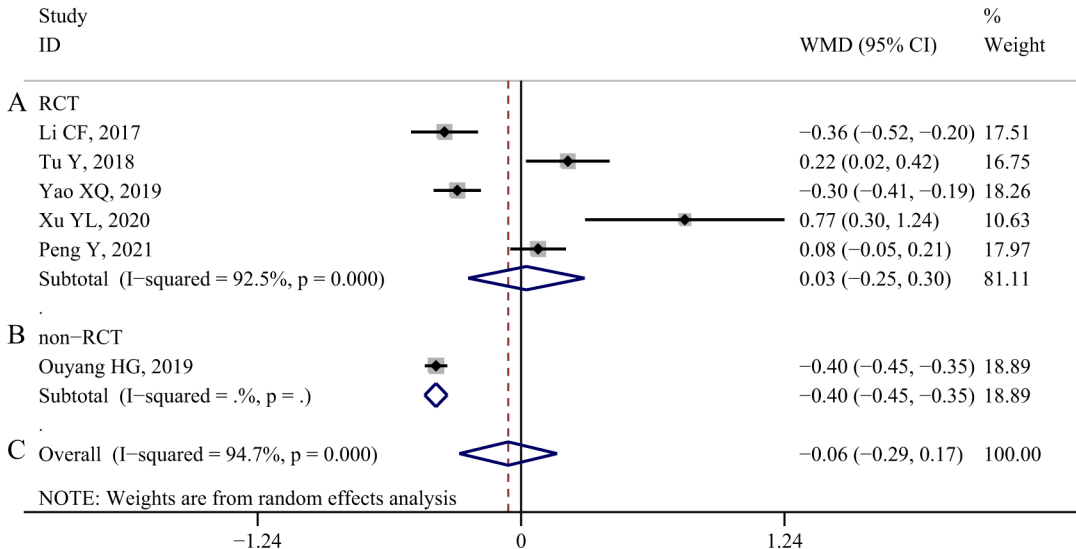

Supplement: Supplementary Materials — Table S1: PRISMA checklists. Table S2: search strategy. Table S3: contents of health literacy assessment tools. Table S4: summary of finding table. Figure S1: usage distribution of health literacy assessment tools. Figure S2: forest plot of FPG by subgroup analysis. Figure S3: forest plot of 2hPG by subgroup analysis. Figure S4: forest plot of HbA1c by subgroup analysis. Figure S5: forest plot of TC by subgroup analysis. Figure S6: forest plot of TG by subgroup analysis. Figure S7: forest plot of LDL-C by subgroup analysis. Figure S8: forest plot of HDL-C by subgroup analysis. Figure S9: sensitivity analysis of intervention effect indexes (A: FPG; B: 2hPG; C: HbA1c; D: TC; E: TG; F: LDL-C; G: HDL-C). [file 1503446.f1.zip › Figure S8-Forest plot of HDL-C by subgroup analysis.pdf]

A

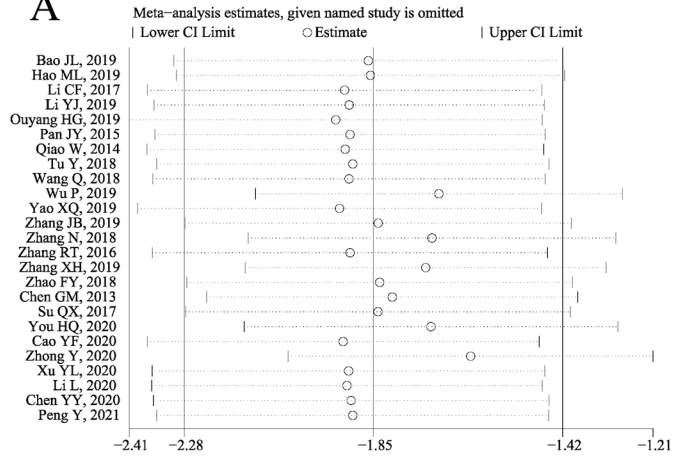

B

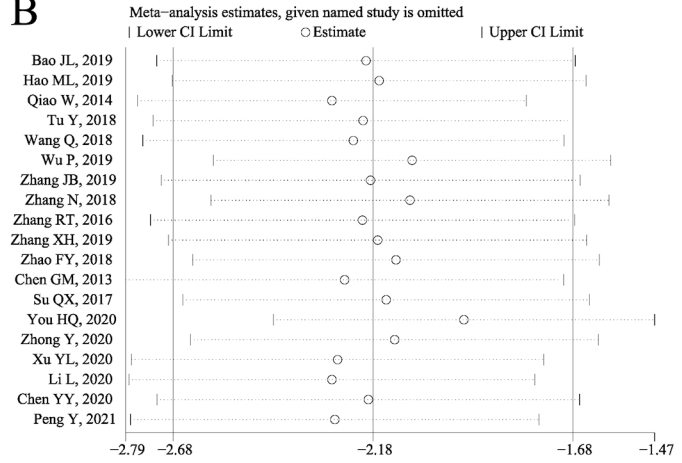

C

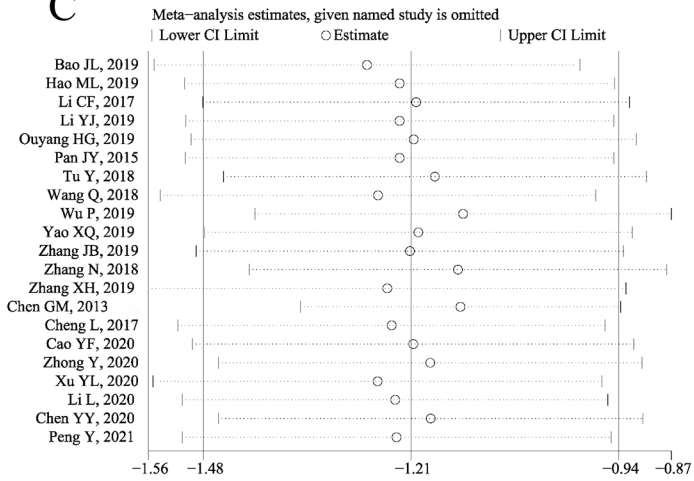

D

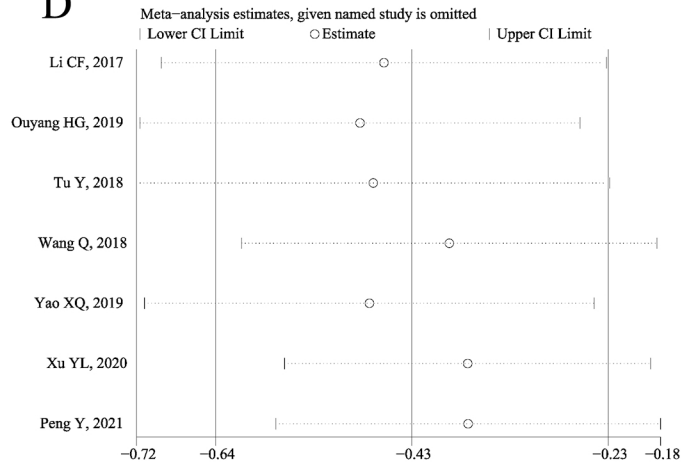

E

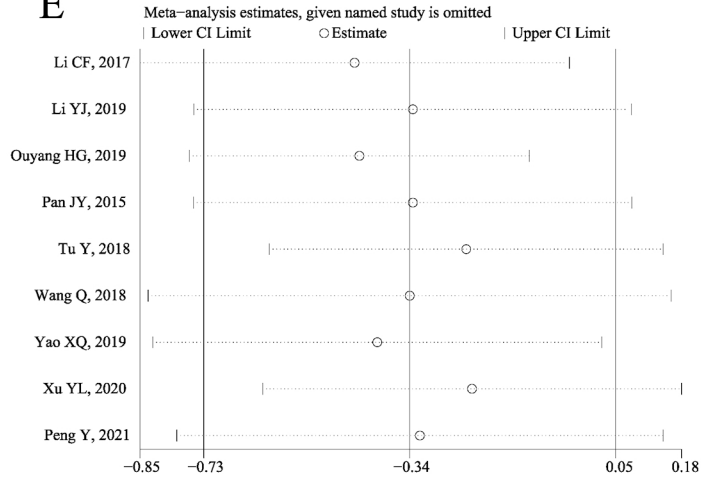

F

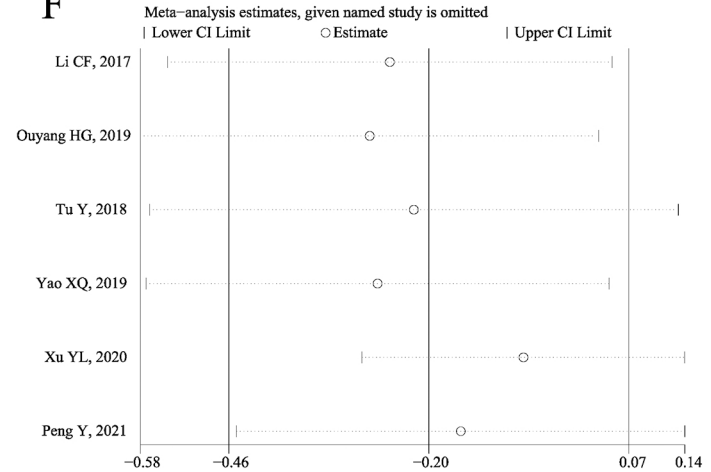

G

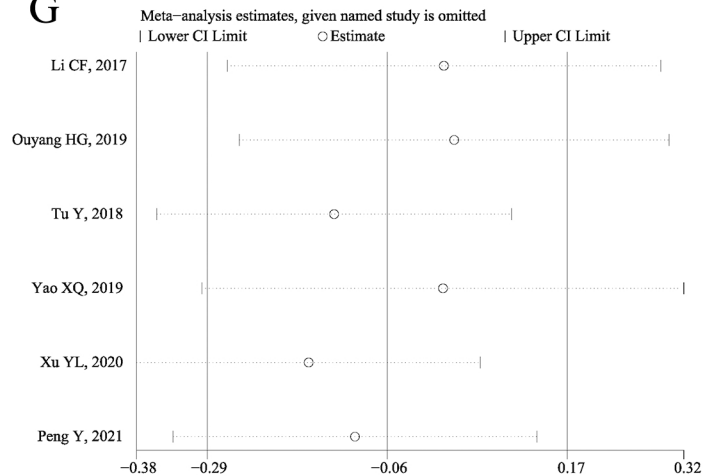

Supplement: Supplementary Materials — Table S1: PRISMA checklists. Table S2: search strategy. Table S3: contents of health literacy assessment tools. Table S4: summary of finding table. Figure S1: usage distribution of health literacy assessment tools. Figure S2: forest plot of FPG by subgroup analysis. Figure S3: forest plot of 2hPG by subgroup analysis. Figure S4: forest plot of HbA1c by subgroup analysis. Figure S5: forest plot of TC by subgroup analysis. Figure S6: forest plot of TG by subgroup analysis. Figure S7: forest plot of LDL-C by subgroup analysis. Figure S8: forest plot of HDL-C by subgroup analysis. Figure S9: sensitivity analysis of intervention effect indexes (A: FPG; B: 2hPG; C: HbA1c; D: TC; E: TG; F: LDL-C; G: HDL-C). [file 1503446.f1.zip › Figure S9-Sensitivity analysis of intervention effect indexes.pdf]
